# Risk of Cataract Incidence in a Cohort of Mayak PA Workers following Chronic Occupational Radiation Exposure

**DOI:** 10.1371/journal.pone.0164357

**Published:** 2016-10-10

**Authors:** Tamara V. Azizova, Evgeny V. Bragin, Nobuyuki Hamada, Maria V. Bannikova

**Affiliations:** 1 Southern Urals Biophysics Institute, Ozyorskoe Shosse 19, Ozyorsk Chelyabinsk Region, Russia; 2 Radiation Safety Research Center, Nuclear Technology Research Laboratory, Central Research Institute of Electric Power Industry (CRIEPI), 2-11-1 Iwado-kita, Komae, Tokyo, Japan; Northwestern University Feinberg School of Medicine, UNITED STATES

## Abstract

This is the first study of cataract incidence in a cohort of Mayak Production Association workers first employed at one of the main facilities in 1948–1982 and followed up till the end of 2008 (22,377 workers). Principal advantages of the study are the large size of the cohort, long-term follow-up and sufficient statistical power, available results of annual eye examinations over the entire follow-up period and detailed information on non-radiation confounders. Individual measured doses from external γ-rays and neutrons used in the analyses were provided by the Mayak Worker Dosimetry System 2008 (MWDS-2008). Relative risk (RR) and excess relative risk (ERR) per unit dose (Gy) were calculated based on maximum likelihood using the AMFIT module of the EPICURE software. The RR of cataract incidence was found to be the highest in workers exposed at doses above 2.0 Gy. A significant linear association of cataract incidence with cumulative dose from external γ-rays was found with ERR/Gy equal to 0.28 (95% confidence intervals: 0.20, 0.37). The results obtained varied slightly with inclusion of additional adjustments for non-radiation factors (smoking index, hypertension, glaucoma and body mass index). Adjusting for the dose from neutrons gave a considerable increase in ERR/Gy for cataract incidence.

## Introduction

Ionizing radiation is known to cause lens opacification [1, 2]. Since Publication 26 of the International Commission on Radiological Protection (ICRP), radiation-induced cataracts have been classified as deterministic effects with a dose threshold depending on the rate of dose delivery (acute, fractionated/protracted, or chronic) [3]. Moreover, thresholds for radiation-induced cataracts and dose limits for the ocular lens have been updated based on available science [4]. The most recent update was made in 2011 [5] when ICRP recommended reducing the threshold from 5 Gy for acute radiation exposure and >8 Gy for fractionated and protracted exposure down to 0.5 Gy independent on radiation dose rate, given that minor opacities progress to vision-impairing cataracts, and considering that the dose threshold for protracted exposure (based on data from Chernobyl clean-up workers [6]) is not greater than that for acute radiation exposure (based on data from atomic bomb survivors [7,8]). Furthermore, ICRP Publication 118 [5] acknowledges uncertainties in risk estimates and dose thresholds for chronic radiation exposure. Thus, studies of cataract risks that aim to clarify a dose-response relationship are very important for radiological protection purposes [4, 9].

The current study was undertaken to investigate the risk of senile cataract incidence in a cohort of Mayak Production Association (PA) workers occupationally exposed to ionizing radiation over a prolonged period, taking into account non-radiation risk factors.

## Materials and Methods

The present record-based epidemiological study did not require any contact with cohort members. The study was reviewed and approved by SUBI Institutional Review Board (IRB). SUBI IRB confirmed that no signed consents were needed from members of the study cohort.

### Characteristics of the Study Cohort and Follow-up

This is a retrospective cohort study. The study cohort included workers employed at Mayak PA—the first nuclear enterprise in Russia located in the Southern Urals in the near vicinity of Ozyorsk city. Mayak PA is a large-scale nuclear enterprise consisting of three main facilities (a plutonium production plant, reactors and a radiochemical plant) and several auxiliary facilities (a mechanical repair plant, liquid gases producing plants, etc), which started its operation in June 1948. The study cohort included all workers first employed at one of its main facilities during 1948–1982, regardless of sex, age, education, nationality, duration of employment and other characteristics (22,377 workers; 25.4% of females). Of these, 43 workers acutely exposed to γ-neutron radiation at a high dose rate that induced acute radiation sickness and 1274 workers lacking medical information due to lost medical records were excluded from the study.

The follow-up of the cohort started from the date of first employment at one of the main Mayak PA facilities, and lasted until the earliest of the following events: date of disease diagnosis; date of death; 31 December 2008 for workers still living in Ozyorsk (residents); date of ‘the last medical information’ for workers-residents with an unknown vital status and for workers who had left Ozyorsk (migrants). Main characteristics of the study cohort are presented in Table 1.

**Table 1:** Characteristics of the study cohort.

Distribution of workers by age at first employment at the main facility						
Age at first employment, years	Males		Females		Both sexes	
	Number	%	Number	%	Number	%
<20	5215	32.83	714	13.79	5929	28.15
20–24	5595	35.23	1840	35.54	7435	35.30
25–29	2464	15.51	1013	19.57	3477	16.51
30–34	1030	6.48	571	11.03	1601	7.60
35–39	752	4.73	555	10.72	1307	6.21
>40	827	5.21	484	9.35	1311	6.23
Total	15883	100	5177	100	21060	100
Distribution of workers by age at cataract diagnosis						
Age at cataract diagnosis, years	Males		Females		Both sexes	
	Number	%	Number	%	Number	%
<40	11	0.43	11	0.69	22	0.53
41–50	172	6.71	53	3.32	225	5.41
51–60	608	23.72	292	18.3	900	21.64
61–70	1338	52.20	869	54.45	2207	53.07
>70	434	16.93	371	23.25	805	19.36
Total	2563	100	1596	100	4159	100
Distribution of workers by age at the end of the follow-up						
Age at the end of the follow-up, years	Males		Females		Both sexes	
	Number	%	Number	%	Number	%
<40	6492	40.87	1548	29.90	8040	38.18
41–50	2043	12.86	436	8.42	2479	11.77
51–60	3301	20.78	979	18.91	4280	20.32
61–70	3134	19.73	1488	28.74	4622	21.95
>70	913	5.75	726	14.02	1639	7.78
Total	15883	100	5177	100	21060	100
Distribution of workers by duration of employment at the main facility						
Duration of employment, years	Males		Females		Both sexes	
	Number	%	Number	%	Number	%
<1	1082	6.81	283	5.47	1365	6.48
1–10	7836	49.34	2613	50.47	10449	49.62
>10	6965	43.85	2281	44.06	9246	43.90
Total	15883	100	5177	100	21060	100
Distribution of workers by cumulative dose from external γ-rays (*H*_p_(10))						
Whole body dose from external γ-rays, Sv	Males		Females		Both sexes	
	Number	%	Number	%	Number	%
<0.25	8291	52.20	3077	59.44	11368	53.98
0.25–0.5	2551	16.06	637	12.30	3188	15.14
0.5–1	2140	13.47	633	12.23	2773	13.17
>1	2901	18.26	830	16.03	3731	17.72
Total	15883	100	5177	100	21060	100
Distribution of workers by cumulative neutron dose absorbed in brain						
Dose from neutrons, Gy	Males		Females		Both sexes	
	Number	%	Number	%	Number	%
<0.001	2054	61.41	408	71.70	2462	62.90
0.001–0.005	1106	33.06	126	22.14	1232	31.48
>0.005	185	5.53	35	6.15	220	5.62
Total	3345	100	569	100	3914	100

More than half of the study cohort workers (55%) were first employed at Mayak PA in the first decade of its operation. The mean age at first employment (± standard error) was 24 ± 0.06 years in males and 27 ± 0.11 years in females; the mean age at study exit was 46 ± 0.14 years in males and 52 ± 0.24 years in females; and the mean duration of employment at the main facilities was 15 ± 0.11 years in males and 14 ± 0.16 years in females.

By the end of the follow-up, vital status was available for 95% of cohort members, of them 53% had deceased while 46.5% were alive.

All disease cases were coded according to the International Classification of Diseases, ninth revision (ICD-9) [10]. All cataracts (ICD-9 code: 366) were identified in the study cohort of Mayak PA workers based on data provided by medical and dosimetry database named ‘Clinic’ [11] regardless of type or etiology– 5222 cases of cataracts (Table 2). Senile cataracts (hereinafter ‘cataracts’, ICD-9 code: 366.1) were the major type of diagnosed cataracts (94%).

**Table 2:** Pattern of cataract cases.

Cause-specific type of cataract	ICD-9 code	Males		Females		Both sexes	
Infantile, juvenile and presenile cataract	366.0	60	1.87	23	1.14	83	1.59
**Senile cataract**	**366.1**	**2986**	**93.28**	**1944**	**96.19**	**4930**	**94.41**
Traumatic cataract	366.2	87	2.72	12	0.59	99	1.90
Cataract secondary to ocular disorders	366.3	40	1.25	29	1.43	69	1.32
Cataract associated with other diseases	366.4	9	0.28	7	0.35	16	0.31
After-cataract	366.5	2	0.06	2	0.10	4	0.08
Other cataract	366.8	5	0.16	3	0.15	8	0.15
Cataract, unspecified	366.9	12	0.37	1	0.05	13	0.25
Total		3201	100	2021	100	5222	100

In the current study, all cases of senile cataracts (4930 cases) were additionally subjected to quality control checks (verification). Archived and current medical records and patients’ medical histories were used as sources of clinical information during verification. To verify cataract cases, the following diagnostic criteria were used: complaints (reduced visual acuity, diplopya, seeing dark spots in one’s vision), manifestation of lens opacities of various shape and localization during eye examinations using various techniques (beam of light passing over an eye, side beam of light, slit-lamp). Intraocular pressure measurements, chronic ocular pathologies, antecedent trauma and surgical interferences were also considered. All Mayak PA workers were subjected to a mandatory health examination prior to employment at one of the main facilities as well as to mandatory annual comprehensive health checks including by ophthalmologists throughout the entire follow-up period. It should be noted that annual eye examinations were performed for each member of the cohort following a standard protocol. Whenever a cataract was diagnosed and reported, an ophthalmologist defined the type of the cataract by location of opacities within the lens (nuclear, cortical, posterior subcapsular, or mixed) and its severity (early, immature, mature, hypermature) in accordance with Russian Federation Health Care System and World Health Organization criteria and standards in force as of the date of diagnostics. As an annual health examination of Mayak PA workers was performed in a special medical center, each worker was examined by one and the same eye specialist over a course of many years (10–15 years).

Eventually 86% of cataracts were confirmed in members of the Mayak PA worker cohort and included in the analysis (4159 cases of senile cataracts). It should be noted that the majority of incident cataracts were registered both in male and female workers aged 61–70 years while the group of workers below age 40 accounted for the least number of cataracts. The mean age of male workers at the date of cataract diagnosis was 63.1 ± 0.15 years while the corresponding age of female workers was 64.8 ± 0.19 years.

Absorbed doses from external γ-rays and neutrons provided by the Mayak Worker Dosimetry System 2008 (MWDS-2008) that was developed in the framework of the Russian-American collaboration were used in the study [12]. MWDS-2008 provides absorbed doses for 18 organs, but the lens dose is unavailable. Individual doses of homogeneous γ-ray exposure absorbed at a point of fixation of a radiation dosimeter on the body at 10 mm depth [*H*_p_(10) dose equivalent] were therefore used in the present study. Moreover, cumulative neutron doses absorbed in brain were also available in MWDS-2008 for workers employed at some manufacturing departments of reactors and exposed to neutrons, and were taken into account. The mean cumulative absorbed doses from external γ-rays were 0.54 ± 0.061 Gy in males and 0.46 ± 0.01 Gy in females, while the mean cumulative neutron doses absorbed in brain were 0.002 ± 0.0001 Gy and 0.002 ± 0.0002 Gy in males and females, respectively.

### Statistical Analysis

Data used in the present analysis were restricted to a period during which time a worker lived in Ozyorsk because no information on diseases, results of annual eye examinations and non-radiation risk factors was available for migrants.

To run the analyses the data were compiled as a multidimensional table (S1 Table).

The analysis provided relative risk (RR) estimates for categories determined for one or several variables while adjusted for other variables. RR was calculated based on maximum likelihood using the AMFIT module of the EPICURE software [13]. 95% confidence intervals (CIs) for RR estimates and *p* values to test the statistical significance were obtained using likelihood-based methods in the AMFIT module.

Incidence rates were intercompared within the study cohort, i.e. an internal control was used, because it was impossible to enroll the compositionally similar control group with available complete medical information and data on risk factors comprised of Ozyorsk residents. This is because only Mayak PA workers were to be medically examined every year during the entire follow-up period and complete medical information including data reported during a preliminary health examination prior to employment of a worker at Mayak PA and all succeeding information reported within the follow-up period is stored in archives only for Mayak PA workers.

The first step was to investigate the influence of non-radiation risk factors on cataract incidence, then the effect of external γ-ray exposure was studied, taking into account non-radiation factors (via stratification).

Along with categorical analyses, trends in incidence with radiation dose were analyzed using Poisson’s regression in the AMFIT module of the EPICURE software. In particular, excess relative risk per unit dose (ERR/Gy) was described with a linear trend with dose from external γ-rays after adjustment (via stratification) for non-radiation factors [sex, attained age (<20, 20–25, …, 80–85, >85 years), age at first employment at Mayak PA (<20, 20–30, 30–40, >40 years), calendar period of birth (<1910, 1910–1920, 1920–1930, 1930–1940, 1940–1950, >1950)]. Thus, the Poisson regression model used was:

$$\lambda =\lambda_{0}(s,aa,fa,bc)\cdot (1+\beta D),$$

where *λ* is cataract incidence rate, *λ*_*0*_ is the background cataract incidence rate, *s* is sex, *aa* is attained age, *fa* is age of first employment at the Mayak PA, *bc* is birth cohort, *β* is ERR/Gy and *D* is cumulative absorbed dose from external γ-rays.

In addition, sensitivity analyses were performed to investigate how extra non-radiation factors, such as hypertension (without hypertension, with hypertension, or hypertension unknown), body mass index (BMI: <normal, normal, >normal, unknown), glaucoma or high myopia preceding a cataract diagnosis, smoking and alcohol consumption status, smoking index (<10, 10–20, >20 pack*years) rather than smoking status, affected the obtained risk estimates.

To investigate the effect from neutron exposure to the estimated risk, we performed a sensitivity analysis. For the analysis, dose from neutrons was considered as a categorical variable and was categorized as follows: <0.01, 0.01–0.05, >0.05, unknown. The adjustment for the dose from neutrons was made via stratification. Namely, the following Poisson regression model was used:

$$\lambda =\lambda_{0}(s,aa,fa,bc,dn)\cdot (1+\beta D),$$

where *dn* is a cumulative dose from neutrons (in Gy units). The workers with unmeasured neutron doses were not excluded from the analysis but were included in an ‘unknown’ category.

We employed such an analysis technique (using the adjustment for neutron dose via stratification) because doses from neutrons were measured only for 18.58% of the study cohort workers, and exclusion of workers with unmeasured neutron doses from the analysis would considerably reduce statistical power of the analysis.

Moreover, the modification of the radiogenic risk of cataract incidence by sex and attained age was studied where heterogeneity and a log-linear trend in ERR/Gy estimate with attained age were assessed. All significance tests were two-sided.

Information on smoking was considered for each worker over the entire follow-up and estimated qualitatively and quantitatively. The qualitative index used included values ‘unknown’, ‘never-smoker’ and ‘ever-smoker’. ‘Never smoker’ denoted a worker who had stated at more than one medical examination within mandatory annual examinations that she/he had never smoked.

Information on alcohol consumption was also considered over the entire follow-up and estimated only as a qualitative parameter with values ‘unknown’, ‘ever drinker’ and ‘never drinker’. ‘Never drinker’ denoted a worker who stated at several interviews during mandatory annual medical examinations that she/he had never drunk alcohol.

Workers with unknown information (e.g. with unknown smoking status or unmeasured neutron doses) were not excluded from the analysis but were included in the category ‘unknown’ for a corresponding factor in each particular case.

Diagnosis of diabetes mellitus was considered over the entire follow-up. In this study, glaucoma and high myopia (hereinafter myopia) manifested prior to cataract diagnosis were taken into account. Myopia was categorized as high if a refractive error was 6 and more diopters.

## Results

By the end of the follow-up (31 December 2008), 4159 cataract cases were registered among Mayak PA workers over 482,217 person-years.

At first, effects of known non-radiation cataract-promoting factors (sex, attained age, smoking, alcohol consumption, diabetes, hypertension, glaucoma, myopia, etc) were analyzed. Table 3 summarizes the RRs of cataract incidence among members of the study worker cohort associated with non-radiation risk factors. No significant differences in RRs of cataract incidence were revealed between males and females. As expected, the RR of cataract incidence increased with attained age of workers. The RR in male workers who had been diagnosed as cataract during the 2006–2008 period was significantly higher than that during the 1996–2005 period. The RR of cataract incidence was affected neither by a calendar period of first employment nor by age at first employment of a worker at a main facility, nor by smoking status, alcohol consumption and diagnosis of diabetes mellitus. Meanwhile the RR of cataract incidence was significantly higher in workers with glaucoma (2.951, 95% CI: 2.470, 3.496) and myopia (2.073, 95% CI: 1.526, 2.749) compared to workers free from these diseases (2.475, 95% CI: 1.823, 3.274 for glaucoma and 1.926, 95% CI 1.384, 2.601 for myopia).

**Table 3:** Relative risk of cataract incidence in the study cohort of workers associated with various factors.

Factor	Males		Females		
	No. of cases	RR (95% CI)	No. of cases	RR (95% CI)	
*RR for females compared to males*					
	2563	1	1596	1.013 (0.948, 1.082)	
*RR for various age groups compared to [65–70] group*					
<30 years	0	–	1	1	
30–35 years	6	0.001 (0.001, 0.003)	3	0.002 (0.001, 0.006)	
35–40 years	5	0.001 (0, 0.002)	7	0.005 (0.002, 0.009)	
40–45 years	48	0.011 (0.008, 0.015)	16	0.01 (0.006, 0.016)	
45–50 years	124	0.032 (0.026, 0.039)	37	0.024 (0.017, 0.033)	
50–55 years	195	0.065 (0.055, 0.077)	87	0.063 (0.049, 0.079)	
55–60 years	412	0.194 (0.172, 0.220)	205	0.182 (0.154, 0.215)	
60–65 years	616	0.443 (0.397, 0.493)	391	0.475 (0.415, 0.544)	
65–70 years	723	1	478	1	
70–75 years	325	1.439 (1.259, 1.641)	274	1.384 (1.19, 1.606)	
75–80 years	97	1.633 (1.309, 2.014)	80	1.186 (0.926, 1.498)	
80–85 years	10	0.949 (0.473, 1.681)	15	0.929 (0.529, 1.505)	
>85 years	2	1.248 (0.206, 3.910)	2	0.746 (0.123, 2.344)	
*RR for workers first employed at one of the main facilities in 1954 and later compared to workers first employed before 1954*					
1948–1953	968	1	766	1	
1954–1958	571	0.973 (0.844, 1.122)	230	0.888 (0.748, 1.053)	
1959–1963	514	1.005 (0.842, 1.202)	187	0.851 (0.645, 1.124)	
1964–1968	218	1.152 (0.904, 1.473)	112	1.101 (0.758, 1.610)	
1969–1972	97	1.048 (0.734, 1.503)	71	1.099 (0.691, 1.761)	
1973–1978	149	1.504 (0.995, 2.297)	178	1.276 (0.780, 2.123)	
1979–1982	46	1.417 (0.796, 2.536)	52	1.189 (0.655, 2.186)	
*RR for various calendar periods of cataract diagnosis compared to [1996–2005] period*					
1948–1955	16	0.550 (0.186, 1.516)	5	0.525 (0.070, 4.145)	
1956–1965	33	0.340 (0.154, 0.720)	11	0.412 (0.114, 1.390)	
1966–1975	158	0.922 (0.576, 1.454)	55	0.905 (0.492, 1.630)	
1976–1985	256	0.871 (0.646, 1.164)	164	0.819 (0.570, 1.165)	
1986–1995	752	0.984 (0.845, 1.144)	520	0.952 (0.793, 1.141)	
1996–2005	937	1	693	1	
2006–2008	411	1.723 (1.487, 1.995)	148	1.084 (0.864, 1.353)	
*RR for groups of age at first employment at the main facility compared to [<20] years group*					
<20 years	566	1	258		1
20–25 years	904	1.073 (0.957, 1.205)	514		0.910 (0.779, 1.067)
25–30 years	439	0.998 (0.868, 1.146)	278		0.771 (0.642, 0.925)
30–35 years	257	0.930 (0.784, 1.100)	167		0.676 (0.550, 0.828)
35–40 years	204	0.877 (0.724, 1.058)	219		0.700 (0.579, 0.845)
>40 years	193	0.816 (0.655, 1.010)	160		0.612 (0.489, 0.761)
*RR for never-smokers compared to ever-smokers*					
Never-smokers	558	0.952 (0.865, 1.047)	1507	1	
Ever-smokers	1995	1	67	1.084 (0.837, 1.379)	
Unknown	10	0.538 (0.269, 0.948)	22	0.749 (0.475, 1.114)	
*RR for smokers and ex-smokers compared to non-smokers*					
Non-smokers	558	1	1507	1	
Ex-smokers	869	1.056 (0.948, 1.177)	35	1.087 (0.759, 1.504)	
Smokers	1126	1.045 (0.944, 1.159)	32	1.080 (0.743, 1.513)	
Unknown	10	0.565 (0.281, 1.000)	22	0.749 (0.475, 1.114)	
*RR for never-drinkers compared to ever-drinkers*					
Never-drinkers	52	0.880 (0.659, 1.148)	847	1	
Ever-drinkers	2329	1	646	1.116 (1.002, 1.243)	
Unknown	182	1.003 (0.858, 1.165)	103	1.116 (0.902, 1.365)	
*RR for workers with diabetes mellitus (DM) compared to workers free from the disease*					
Free from DM	2399	1	3824	1	
Diagnosed with DM	164	0.870 (0.740, 1.017)	335	0.944 (0.842, 1.054)	
*RR for workers diagnosed with glaucoma compared to workers free from the disease*					
Free from glaucoma	2421	1	1545	1	
**Diagnosed with glaucoma**	**142**	**2.951 (2.470, 3.496)**	**51**	**2.073 (1.526, 2.749)**	
*RR for workers diagnosed with high myopia compared to workers free from the disease*					
Free from high myopia	2516	1	1554	1	
**Diagnosed with myopia**	**47**	**2.475 (1.823, 3.274)**	**42**	**1.926 (1.384, 2.601)**	

Table 4 shows the RRs of cataract incidence associated with cumulative dose from external γ-rays. Risk of cataract incidence increased with dose from external γ-rays and was significant for all dose categories when compared to the reference category (0–0.25 Sv). RR of cataract incidence was highest in the category of workers exposed to external γ-rays at doses above 2 Sv (1.61, 95% CIs: 1.41, 1.83).

**Table 4:** RRs of cataract incidence associated with cumulative dose from external γ-rays.

Cumulative external γ dose (Sv), range	Mean cumulative external γ dose (Sv)	Person-years of the follow-up	Number of cataract cases	RR (95% CI)	p-value
(0–0.25)	0.08	255036	1631	1	< 0.001
(0.25–0.50)	0.36	69097.1	702	1.23 (1.11, 1.35)	0.0513
(0.50–0.75)	0.62	35678.2	365	1.13 (1.00, 1.28)	< 0.001
(0.75–1.00)	0.87	25915	321	1.38 (1.21, 1.57)	< 0.001
(1.00–1.25)	1.12	18191.8	224	1.43 (1.23, 1.66)	< 0.001
(1.25–1.50)	1.37	15147.2	217	1.57 (1.34, 1.83)	< 0.001
(1.50–2.00)	1.73	20066.3	296	1.59 (1.39, 1.83)	< 0.001
**≥2.00**	**2.67**	**25498**	**387**	**1.61 (1.41, 1.83)**	< 0.001

**Fig 1:**
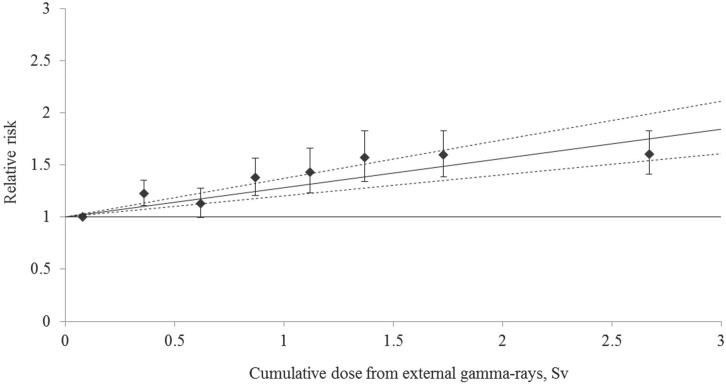
Cataract incidence risk associated with cumulative dose from external γ-rays. Error bars present 95% confidence intervals (CIs).

Table 5 shows the risk of cataract incidence associated with dose from external γ-rays based on the linear model. Based on the linear model it was found that an increased cataract incidence was significantly linearly associated with increasing cumulative dose from external γ-rays; ERR/Gy was 0.281 (95% CI: 0.20, 0.37) (Table 5 and Fig 1).

**Table 5:** Risk of cataract incidence associated with dose from external γ-rays.

Analysis	ERR/Gy (95% CI)
**Baseline analysis, 0-year lag**	**0.28 (0.20, 0.37)**
Baseline analysis adjusted for smoking and alcohol, 0-year lag	
	0.29 (0.20, 0.38)
*Additional stratification (0-year lag)*:	
By hypertension	0.26 (0.18, 0.35)
By body mass index	0.24 (0.17, 0.33)
By glaucoma	0.29 (0.21, 0.38)
By myopia	0.28 (0.21, 0.37)
**By neutron dose in the brain**	**0.31 (0.22, 0.40)**
By smoking index	0.26 (0.18, 0.35)
*Analysis (0-year lag) restricted to workers*:	
Males	0.23 (0.15, 0.34)
Females	0.40 (0.24, 0.59)
	*p* = 0.09
Attained age	
<40 years	0.5 (n/a, 7.98)
40–49 years	0.54 (0.16, 1.24)
50–59 years	0.22 (0.08, 0.42)
60–69 years	0.26 (0.16, 0.38)
>70+ years	0.35 (0.17, 0.59)
	*P*_*1*_ >0.50
	*P*_*2*_ >0.50
n/a, not available	

P1 –Test for heterogeneity between groups of workers of different attained age.

P2 –Test for a log-linear trend in the ERR/Gy with attained age.

Additional stratification by smoking and alcohol consumption had no effect on the result. ERR/Gy for cataract incidence decreased modestly after adjustment for hypertension, body mass index and smoking index while adjusting for glaucoma slightly increased the risk. After an adjustment for cumulative neutron dose absorbed in brain was included, the ERR/Gy for cataract incidence increased (0.31, 95% CIs: 0.22, 0.40). The ERR/Gy for cataract incidence was significantly increased both in male and female workers of the study cohort; however, no significant difference was revealed between these estimates (*p* = 0.09). ERRs/Gy gained statistical significance in all age categories except for the group of workers younger than 40 years, but again, the difference among the age categories was insignificant (*p* >0.5). Risk of cataract incidence tended to decrease with increasing age, but this trend was not significant either (*p* >0.5).

## Discussion

This is the first study of cataract incidence in a cohort of Mayak PA workers first employed at one of the main facilities in 1948–1982 and followed-up until the end of 2008.

Here we have demonstrated that RR of senile cataract incidence in the cohort of Mayak PA workers occupationally exposed to ionizing radiation over a prolonged period was significantly associated with both non-radiation (attained age, ocular co-morbidity) and radiation (external γ-ray and neutron exposure) risk factors. As anticipated given the role of the biology of aging in the pathology under the study, the RR of cataract incidence increased with attained age of the study cohort workers. However, a series of other factors related to cataract development have been identified in numerous epidemiological and clinical studies: solar (and other sources) UV and infrared radiation, diabetes mellitus, hypertension, obesity, smoking, long-term treatment with corticosteroids, statin treatment, substitutive hormonotherapy, precedent trauma and ocular inflammatory diseases, abusive drinking behavior, high myopia and inherited predisposition [14]. At the same time, data on effects of various non-radiation factors (e.g., sex, smoking, and alcohol consumption) on cataract development are inconsistent [15–21].

The present study failed to observe a significant increase in the risk of cataract incidence in females compared to males (RR = 1.013, 95% CI: 0.948, 1.082). Similarly, to other studies [18], no significant difference in risks was observed among smokers, non-smokers and ex-smokers in the present study of Mayak PA worker cohort. The effect of alcohol consumption on cataractogenesis is still unclear. Some studies [19] found increased risks of cataracts including surgically removed cataracts among alcohol abusers whilst other studies [20] found a protective effect of wine drinking for some types of cataracts. This study found no significant effect of alcohol consumption status on cataract incidence. Meanwhile, the revealed increased risk of cataracts in the study cohort of Mayak PA workers diagnosed with glaucoma and/or high myopia agrees well with results of other studies [21].

Standardized incidence rates for Mayak PA workers first employed at one of the main facilities during 1948–1982 were 731.5 ± 14.5 per 100,000 in males and 1210 ± 30.3 per 100,000 in females [22]. These rates are consistent with the national average incidence rate (in Russia) [23].

ERRs/Gy obtained in the present study (1.28 95% CIs: 1.20, 1.37) show good agreement with results of the Japanese cohort study of A-bomb survivors, in the first instance, with those reported for risks of total cataract regardless of its subtype. ERRs/Gy in the Adult Health Study were 1.3 (95% CIs: 1.1, 1.5) for surgically removed cataracts [24] and 1.06 (95% CIs: 1.01, 1.11) for total cataracts [7]. The risk estimated for cataract incidence in the present study was markedly lower than those reported for the cohort of Chernobyl clean-up workers which was 1.6 (95% CIs: 1.2, 2.3) [6] and found for surgically removed cataracts in the US radiologists which was 2.5 (95% CIs: <1.07, 7.4) [25].

A recent critical review of all available studies of cataract incidence following different exposure scenarios [26] concluded that there have thus far been insufficient data to provide a conclusive evidence of cataract incidence risk association with radiation dose. Higher radiation doses have a greater cataract incidence, but the shape of the dose response curve, especially at low doses is not clear from the available data, and the underlying basic mechanisms of action remain incompletely understood. The performed studies of cataract incidence are heterogeneous in their design, population or cohort size under investigation, cataract diagnostics techniques, statistical methods, etc. Exact date of cataract diagnosis was not available in many studies, and main cataract-promoting non-radiation factors were not taken into account.

The present study showed that ERRs/Gy varied with inclusion of some confounding factors via stratification (smoking index, body mass index, hypertension). Inclusion of an adjustment for neutron dose provided an increased risk estimate.

Principal advantages of the study of cataract incidence in the cohort of Mayak PA workers were the large size of the cohort, long-term follow up period, and sufficient statistical power. It should be particularly emphasized that an ophthalmologist appointment and an ocular examination were mandatory clinical procedures as part of annual health examinations performed for all workers of the study cohort. Moreover, detailed information on non-radiation confounders including their quantitative characteristics was obtained for the Mayak PA worker cohort.

The main limitation of the present study is the lack of specific information on radiation doses to eye lens within MWDS-2008 dosimetry system [12]. However, detailed histories of occupational exposures, individual measurements of external γ-ray doses, explicit characteristics of exposure scenarios and data on job position will enable future reconstruction of eye lens doses.

Despite the fact that the number of studies on cataract risks has been constantly increasing in recent years, some challenges in cataract type-specific risk assessment still exist, especially for workers chronically exposed to radiation at low dose rates. Available epidemiological studies suggest the increase in incidence of posterior subcapsular or cortical cataracts with radiation dose [26, 27]. Meanwhile, the most common senile cataracts are nuclear and cortical cataracts. In the study Mayak PA worker cohort, cortical (48.26%) and nuclear (31.39%) cataracts were the most prevailing types of senile cataract. Posterior subcapsular cataracts accounted for 19.3% of the disease cases. The next stage of the study aims to assess risks and dose-response relationships for different types of cataracts taking into account non-radiation risk factors and using reconstructed eye lens radiation doses.

It is not only the dose response that is of importance but mechanisms of cataract development following prolonged low-dose-rate radiation exposure as well [28, 29]. The cohort of Mayak PA workers is one among few cohorts with biological samples collected from its members (e.g., blood, DNA) in hand. The collection of cells of eye lens removed during cataract surgeries has been initiated, which facilitates future studies aiming to identify markers of radiation—induced health effects and to better understand mechanisms of cataractogenesis in radiation-exposed individuals.

## Conclusions

Cataract incidence in the cohort of Mayak PA workers first employed in 1948–1982 and followed up till the end of 2008 was shown to be affected by non-radiation (attained age, glaucoma, high myopia) and radiation (external γ-ray and neutron exposure) factors. The RR of cataract incidence was found to be the highest in workers exposed at doses above 2.0 Gy. A significant linear association of increased cataract incidence with increasing cumulative dose from external γ-rays was revealed with ERR/Gy equal to 0.28 (95% CI: 0.20, 0.37). The obtained result slightly varied with inclusion of additional adjustments for non-radiation factors (smoking index, hypertension, glaucoma, body mass index). Adjusting for the dose from neutrons gave a considerable increase in ERR/Gy for cataract incidence.

## Supporting Information

S1 Table Description of variables used for the analysis. (DOCX)
