# Supplementary material for: Risk of Cataract Incidence in a Cohort of Mayak PA Workers following Chronic Occupational Radiation Exposure
Source: PLoS One. 2016 Oct 10;11(10):e0164357. doi: 10.1371/journal.pone.0164357 (PMC5056693; doi:10.1371/journal.pone.0164357)
Supplement: S1 Table — (DOCX) [file pone.0164357.s001.docx]

**S1 Table**. Description of variables used for the analysis

| **Variable type** | **Name of variable** | **Description of variable** | |
| --- | --- | --- | --- |
| Fixed | Sex | 1:Males | 2:Females |
| Fixed | Calendar period of birth | 1:<1910  2:1910–1919  3:1920–1929 | 4:1930–1939  5:1940–1949  6:1950+ |
| Fixed | First year of employment | 1:1948–1953  2:1954–1958  3:1959–1963  4:1964–1968 | 5:1969–1972  6:1973–1978  7:1979–1982 |
| Fixed | Age at first employment | 1:<20  2:20–24  3:25–29 | 4:30–34  5:35–39  6:40+ |
| Fixed | Hypertension | 1: unknown  2: without hypertension | 3: hypertension |
| Fixed | Body mass index | 1: unknown  2: <normal | 3: normal  4: >normal |
| Fixed | Glaucoma | 1: Free from glaucoma  2: Diagnosed with glaucoma |  |
| Fixed | High myopia | 1: Free from high myopia  2: Diagnosed with myopia |  |
| Fixed | Diabetes | 1: Free from diabetes  2: Diagnosed with diabetes |  |
| Fixed | Smoking status | 1: Non-smokers  2: Ex-smokers | 3: Smokers  4: unknown |
| Fixed | Alcohol consumption | 1: Never-drinkers  2: Ever-drinkers | 3: unknown |
| Time varying | Attained age categories | 1: 15–20  2: 20–25  3: 25–30  4: 30–35  5: 35–40  6: 40–45  7: 45–50  8: 50–55 | 9: 55–60  10: 60–65  11: 65–70  12: 70–75  13: 75–80  14: 80–85  15: 85+ |
| Time varying | Calendar time categories | 1: 1947–1950  2: 1951–1955  3: 1956–1960  4: 1961–1965  5: 1966–1970  6: 1971–1975  7: 1976–1980 | 8: 1981–1985  9: 1986-1990  10: 1991–1995  11: 1996–2000  12: 2001–2005  13: 2006–2008 |
| Time dependent | Smoking index (pack*years) categories | 1: =0 (non-smokers)  2: unknown  3: <10 | 4: 10–20  5: >20 |
| Time dependent | Cumulative external γ dose (Sv) categories | 1: 0–0.25  2: 0.25–0.50  3: 0.50–0.75  4: 0.75–1.00 | 5: 1.00–1.25  6: 1.25–1.50  7: 1.50–2.00  8: >=2.00 |
| Time dependent | Dose from neutrons (Gy) categories | 1: unknown  2: <0.01  3: 0.01–0.005 | 4: >0.05 |
| Calculated | Attained age | Person-year weighted mean attained age in years | |
|  | Cumulative external γ dose, Sv | Person-year weighted mean cumulative gamma dose | |
|  | PYR | Person years at risk | |
| Case counts | Cataract | Cataracts (ICD-9: 366.0) | |
